# 1816. Effectiveness of Cephalexin and Clavulanic Acid Fixed-Dose Combination (FDC), Amoxicillin and Clavulanic Acid FDC, and Cefuroxime in Indian Patients with Dental Infections: Subset Analysis of Patients with and without Comorbidities from Real-World Retrospective Electronic Medical Records

**DOI:** 10.1093/ofid/ofad500.1645

**Published:** 2023-11-27

**Authors:** Archana Karadkhele, Rupali Jangid, Saleha Rehman, Raman Gupta, Venugopal Madhusudana, Shruti Dharmadhikari, Chintan Khandhedia, Neeraj Markandeywar, Amey Mane, Suyog Mehta

**Affiliations:** Sun Pharma Laboratories Limited, Mumbai, Maharashtra, India; THB c/o Sekhmet Technologies Private Limited, Gurugram, Haryana, India; THB c/o Sekhmet Technologies Private Limited, Gurugram, Haryana, India; THB c/o Sekhmet Technologies Private Limited, Gurugram, Haryana, India; THB c/o Sekhmet Technologies Private Limited, Gurugram, Haryana, India; Sun Pharma Laboratories Limied, Mumbai, Maharashtra, India; Sun Pharma Laboratories Limited, Mumbai, Maharashtra, India; Sun Pharma Laboratories Limited, Mumbai, Maharashtra, India; Sun Pharma Laboratories Limited, Mumbai, Maharashtra, India; Sun Pharma Laboratories Limited, Mumbai, Maharashtra, India

## Abstract

**Background:**

Globally 20-50% population suffer from periodontal diseases. Bidirectional link exists between periodontal diseases and comorbidities. Limited evidence is available on antibiotic effectiveness in patients of dental infections with comorbidities.

**Methods:**

This retrospective, multi-centric, observational electronic medical record study gathered real world Indian evidence to assess effectiveness of cephalexin-clavulanic acid (cephalexin CV) fixed-dose combination (FDC) compared to amoxicillin-clavulanic acid (amoxicillin CV) FDC and cefuroxime in adults with dental infections. Following data represents effectiveness in subset of patients with and without comorbidity.

**Results:**

Overall, 89% (316/355) patients were without comorbidity [51% (160/316) in cephalexin CV, 22% (70/316) in cefuroxime and 27% (86/316) in amoxicillin CV]; while 11% (39/355) patients had comorbidities [36% (14/39) in cephalexin CV, 44% (17/39) in cefuroxime and 20% (8/39) in amoxycillin-clavulanic acid].

Time to overall clinical improvement was less in patients without comorbidities compared to those with comorbidities (4.67±2.02 days vs 5.41±3.10 days; p= 0.154).

In patients without comorbidity, 98.13% (157/160), 100% (70/70), and 97.67% (84/86) showed overall clinical improvement within 10 days, in cephalexin CV, cefuroxime, amoxicillin CV groups, respectively.

In patients with any comorbidity, 100% (14/14) patients showed improvement in cephalexin CV against 94% (16/17) in cefuroxime and 87% (7/8) in amoxicillin CV group within 10 days (p >0.05).

Overall clinical improvement in patients with and without comorbidities on Day 5 and 7 is presented in Table. In metabolic and cardiovascular disorders subsets, 100% patients showed improvement within 10 days in cephalexin CV group, while 92% (12/13) and 100% (5/5), respectively in cefuroxime group; and 88% (7/8) and 67% (2/3), respectively in amoxicillin CV (p >0.05).

**Figure:**
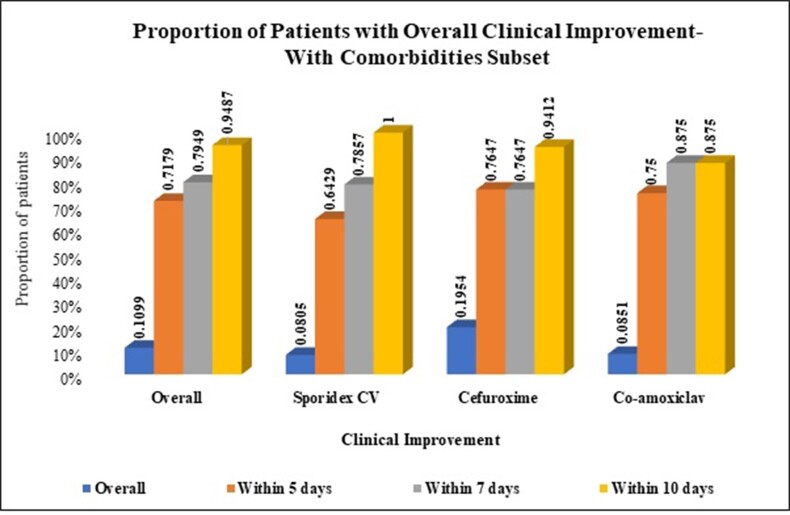

**Figure:**
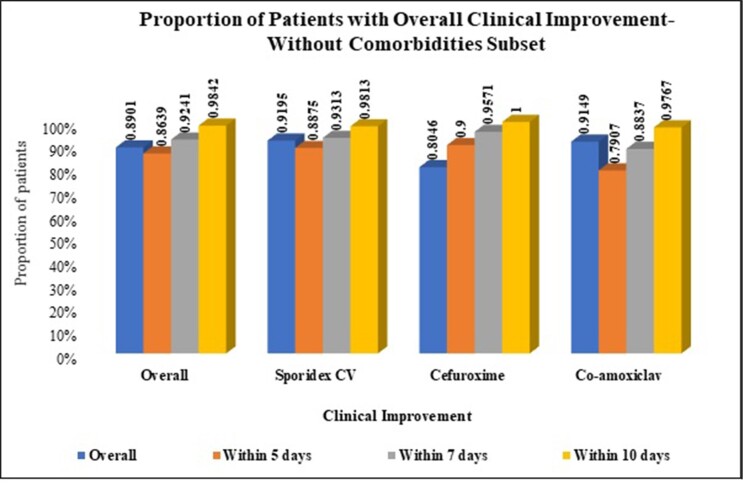

**Figure:**
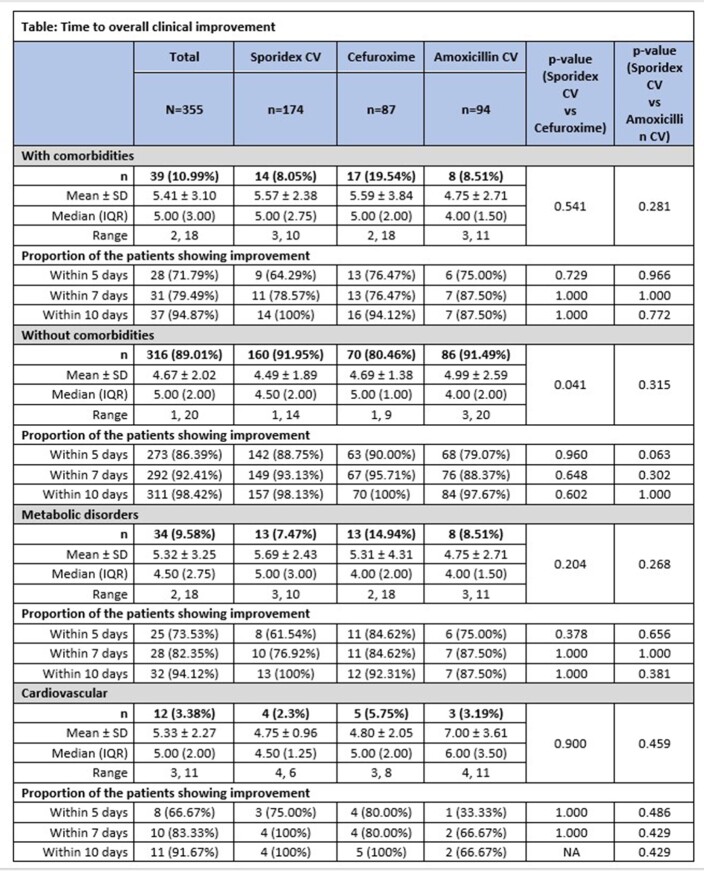

**Conclusion:**

In patients with dental infections, presence of comorbidities delays overall clinical improvement. All patients with comorbidity treated with cephalexin CV showed overall clinical improvement within 10 days of treatment. No significant difference was observed between treatments for overall clinical improvement.

**Disclosures:**

**Archana Karadkhele, MBA**, Sun Pharma Laboratories Limited: Full time employee **Shruti Dharmadhikari, MSc**, Sun Pharma Laboratories Limited: Full time employee **Chintan Khandhedia, MD**, Sun Pharma Laboratories Limited: Full time employee **Neeraj Markandeywar, MD**, Sun Pharma Laboratories Limited: Full time employee **Amey Mane, MD**, Sun Pharma Laboratories Limited: Full time employee **Suyog Mehta, MD**, Sun Pharma Laboratories Limited: Full time employee

